# Environmental and Historical Determinants of Patterns of Genetic Differentiation in Wild Soybean (*Glycine soja* Sieb. et Zucc)

**DOI:** 10.1038/srep22795

**Published:** 2016-03-08

**Authors:** Shui-Lian He, Yun-Sheng Wang, De-Zhu Li, Ting-Shuang Yi

**Affiliations:** 1 Plant Germplasm and Genomics Center, Germplasm Bank of Wild Species, Kunming Institute of Botany, Chinese Academy of Sciences, Kunming, 650201, China; 2 College of Horticulture and Landscape, Yunnan Agricultural University, Kunming, 650201, China; 3 School of Environmental and life Science, Kaili University, Kaili, 650201, China

## Abstract

Wild soybean, the direct progenitor of cultivated soybean, inhabits a wide distribution range across the mainland of East Asia and the Japanese archipelago. A multidisciplinary approach combining analyses of population genetics based on 20 nuclear microsatellites and one plastid locus were applied to reveal the genetic variation of wild soybean, and the contributions of geographical, environmental factors and historic climatic change on its patterns of genetic differentiation. High genetic diversity and significant genetic differentiation were revealed in wild soybean. Wild soybean was inferred to be limited to southern and central China during the Last Glacial Maximum (LGM) and experienced large-scale post-LGM range expansion into northern East Asia. A substantial northward range shift has been predicted to occur by the 2080s. A stronger effect of isolation by environment (IBE) versus isolation by geographical distance (IBD) was found for genetic differentiation in wild soybean, which suggested that environmental factors were responsible for the adaptive eco-geographical differentiation. This study indicated that IBE and historical climatic change together shaped patterns of genetic variation and differentiation of wild soybean. Different conservation measures should be implemented on different populations according to their adaptive potential to future changes in climate and human-induced environmental changes.

Genetic diversity is key for a species to survive and adapt to changing environments^1^, and one fundamental task in biology is to elucidate the underlying mechanisms of the origin and maintenance of genetic variation^2^. The detailed information of genetic variation could be applied to reveal the demographic history and population structure of a species^3^,^4^,^5^ and the underlying genetic mechanisms of local adaptation and evolutionary changes^6^,^7^,^8^,^9^,^10^.

Two processes are widely acknowledged to be major drivers of genetic differentiation: isolation by geographical distance (IBD) and isolation by environment (IBE)^11^,^12^,^13^,^14^. Under the IBD scenario, the amount of gene flow is mainly restricted by geographical distance, and genetic differentiation is expected to increase according to the distance between populations^15^. However, under IBE, the fitness of immigrants or hybrids between adjacent populations that adapt to distinct environments may be reduced by natural selection^12^, which will facilitate or maintain genetic divergence^16^, and the genetic differentiation between populations is correlated to the influence of environmental variables on gene flow^11^,^17^. Geographical processes may influence the genetic structure of a population at large spatial scales, whereas ecological processes may influence the genetic structure of a population at small spatial scales^18^,^19^. In addition to the above contemporary geographic and environmental factors, shifting environmental conditions over time may be crucial factors for genetic differentiation^20^. Recent studies have considered the relative contribution of IBD and IBE on genetic variation at a species-wide scale^15^,^21^,^22^,^23^,^24^,^25^. However, few studies have jointly considered the relative importance of the contemporary IBD and IBE and historical climate change on genetic variation.

East Asia exhibits high topographic complexity and climate variability and harbours high levels of diversity of temperate plant species^26^. Although this region has never been directly impacted by extensive and unified ice-sheets^27^, it experienced severe climatic oscillations throughout the Quaternary, with dramatic effects on the evolution and distribution of both plants and animals^28^. The Japanese Archipelago was repeatedly connected with East China via the exposed wide stretches of continental shelf of the East China Sea (ECS) during glacial periods^29^. Simulated paleovegetation reconstructions suggest that a band of warm temperate deciduous forest extending on this land-bridge across the ECS connected the presently isolated temperate forests of China and Japan during the Last Glacial Maximum^28^. As one of the earliest and most human-influenced regions, the local biological diversity has been significantly affected by overexploitation and intensive agriculture and land use practice. Wild soybean is the direct progenitor of cultivated soybean (*Glycine max* (Linnaeus) Merrill), which is widely distributed in East Asia, including major parts of China, the Japanese archipelago, the Korean peninsula and the Russian Far East^30^. Wild soybean usually grows in moist habitats near freshwater resources from the sea to 2650 m above sea level, in subtropical (southward to 24°N) to subfrigid zones (northward to 53°N). It also occurs in various habitats in salty lands and seasonally dry areas. Wild soybean is mainly distributed in open habitats with frequent human activities, and its distribution region has been significantly fragmented and reduced by land exploitation and utilization. This species is even extinct in the wild in some regions and has been listed as a rare and endangered plant in China^31^. Wild soybean thus supplies a good model to address the relative contribution of IBD, IBE and historical climatic change on its genetic variation and to explore conservation measures that integrate present genetic variation and changes in distribution under historical climatic change.

Various molecular markers, such as RAPD, SSRs and gene sequences, have been applied to address the population structure of wild soybean^31^,^32^,^33^,^34^,^35^. High intra- and inter-population genetic variation has been revealed^34^,^36^,^37^,^38^,^39^. Three evolutionary significant units (ESUs) were revealed by some studies: Northeast, Southeast and Yellow River Valley^40^,^41^, whereas other recent studies tend to combine the Northeast and Southeast into one ESU^31^,^39^. Some recent studies found a correlation between genetic distance and geographical distance^33^,^42^, which indicates IBD is involved in the genetic differentiation of wild soybean. However, the influence of environmental variables on the genetic divergence (namely IBE) of this species has not been addressed.

Applying 20 nuclear Simple Sequence Repeat markers (nSSRs) and one cpDNA locus (*trnQ-rps16*) and a multidisciplinary approach combining population genetic analyses, ecological niche modelling, a Bayesian skyline plot, a Mantel test and a principle components analysis, the major aims of this study were (i) to detect the genetic variation of wild soybean; (ii) to elucidate the relative contribution of geographical, environmental and historical effects on the distribution and genetic differentiation of wild soybean; and (iii) to predict the fate of wild soybean as it is confronted with rapid environmental and climate changes and to provide information to design effective conservation and management strategies for wild soybean.

## Results

### Genetic variation and structure of wildsoybean

For microsatellite data from 43 populations, a null homozygote was found in all the loci with low frequencies (<5%). All 20 loci were polymorphic (Table S1). The polymorphism information content (PIC) for each locus ranges from 0.764 to 0.940, with an average of 0.883 (Table S1). Genetic diversity parameters are presented in Table 1. Alleles in wild soybean are rich, with an average of 3.3 alleles per population. The mean expected heterozygosity (*H*_E_) is 0.426 over all loci for each population ranging from 0.018 to 0.797. High differentiation was revealed by the global *F*_ST_ value (0.509), which indicated significant population genetic structure existed in wild soybean populations. The AMOVA analysis of nSSRs revealed that 6.0% of the genetic variation was due to the genetic distance between the two clusters, 46.7% was due to populations within clusters and 47.3% was due to individuals within populations (Table 2).

**Figure 1:**
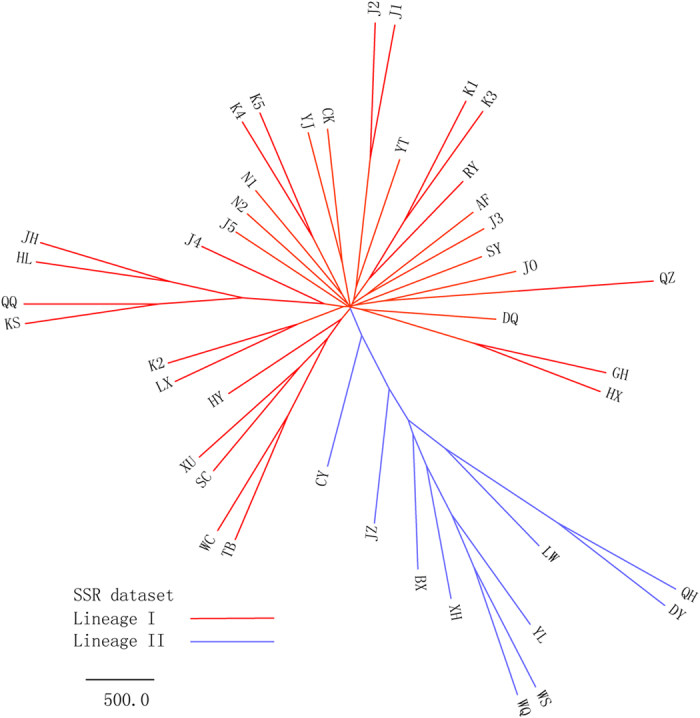
Clustering analysis of wild soybean populations based on UPGMA.

**Figure 2:**
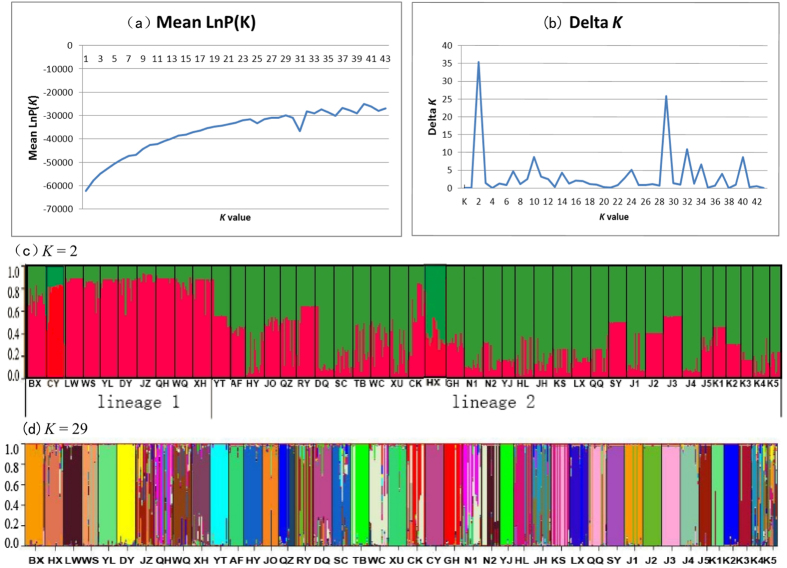
Inferred population structure based on 43 populations and 20 nSSRs of wild soybean. (**a**) Genetic structure of wild soybean inferred from the admixture model (*K* = 2); (**b**) Genetic structure of wild soybean inferred from the admixture model (*K* = 29).

The UPGMA tree based on Nei’s standard genetic distance is shown in Fig. 1. The 43 wild soybean populations were resolved into two lineages: lineage I was formed by eight populations from the Yellow River and Huai River valley in addition to population CY from Tibet; lineage II was formed by the remaining 34 populations. Populations from Japan and Korea did not form independent lineages. The MCMC structure reconstruction of nSSRs is shown in Fig. 2. ΔK showed extremely high values at *K* = 2 and 29 when Evanno’s *ad hoc* estimator of the actual number of groups was used (Fig. 2b). When *K* = 2, two clusters were separated that largely correspond to those of the UPGMA analyses (Fig. 2c). Figure 2d showed the inferred clusters with *K* = 29 and revealed uniform and admixed populations. For example, a comparison of the K5 and J2 populations showed a low level of genetic similarity within the site in the former population, indicating population admixture, whereas the latter population was very uniform and showed only minor differences between microsites.

**Figure 3:**
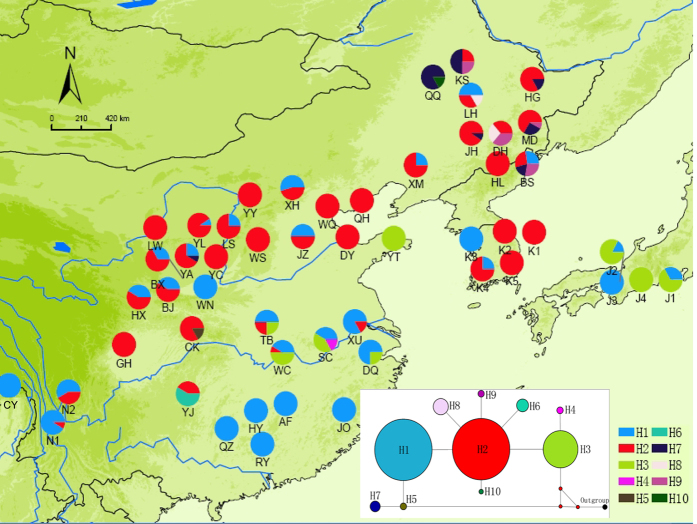
Haplotype distributions of wild soybean populations. (ArcMap v9.3 and NETWORK v4.6: http://www.fluxus-engineering.com/sharepub.htm#a10).

A total of 10 different cpDNA haplotypes (H1–H10) were identified based on 9 polymorphism sites detected from *trnQ-rps16*. Different haplotypes had quite different frequencies: H1 (35.2%) and H2 (46.1%) were two most common and widespread haplotypes, which were found in most populations of wild soybean. However, each of H5, H6, H7 and H10 was an endemic haplotype, which was found in only one population (Fig. 3). The ancestral haplotype could not be identified.

The Bayesian Skyline plots indicated that the population size of wild soybean has experienced a rapid increase following a long period of relative stability. This rapid increase was inferred to occur after the last glacial maximum and at the beginning of the warming period in the early Holocene (15,000 years before present, Fig. S1).

### Relationships between genetic variation and environmental versus geographical factors

A Mantel test revealed a significant correlation between genetic distance and environmental distance (*r* = 0.233, *P* = 0.002), but no significant correlation exists between genetic distance and geographical distance (*r* = −0.016, *P* = 0.341). When geographical factors were controlled, a partial Mantel test also revealed isolation by environmental distance (*r* = 0.232, *P* = 0.001). Where as when environmental factors were controlled, we could not detect significant correlations between genetic differentiation and geographical distance (*r* = −0.002, *P* = 0.508). The MMRR analysis suggested that the environment factors had a higher regression coefficient, whereas the effects of geographic distance were not significant (geographic distance: *β* = 0.005677, *P* = 0.2939; environment distance: *β* = 0.205233, *P* = 0.0249; Table 3).

### LGM, Present and future distribution of wild soybean

**Figure 4:**
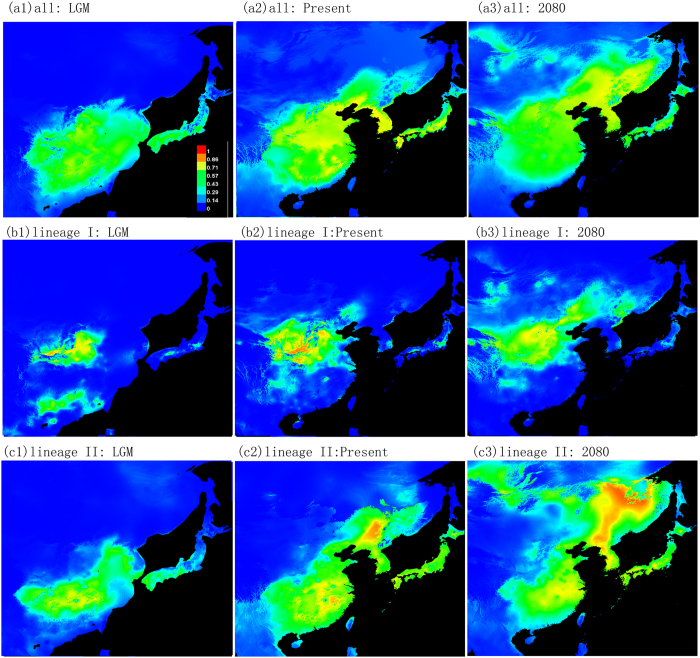
Potential distributions as the probability of occurrence for wild soybean. Suitability values indicate logistic probabilities ranging from 0–1, with increasingly darker shades of red with increasing habitat suitability. (**a**) All populations; (**b**) Lineage I; (**c**) Lineage II (MAXENT v3.3.3 & Adobe illustrator CS2).

All models performed well with AUC values >0.9 (n = 10 replicate model runs) suggesting a high fit of the model^43^. The predicted distribution of wild soybean (Fig. 4a2) is consistent with the observed present distribution, indicating that the distribution is restricted by environmental factors. A Jackknife of the regularized training gain revealed that bio2, bio3 and bio15 made only small contributions to model development. However, bio1, bio4, bio5 and bio13 contributed the most to model development. Over all, temperature had a greater influence on wild soybean than precipitation (Fig. S2). The distribution of the LGM based on MIROC (Fig. 4a1) differed substantially from the present. The estimated distribution of wild soybean during the LGM was restricted to southern and central China. No suitable habitat found in northeastern China and northern Honshu in Japan. Both lineages I and II experienced a northward shift after the LGM; however, lineage I has expanded on a much smaller scale than lineage II. Lineage II has most probably dispersed into northern and northeastern China, Korea, and northern Japan from its southern refugia. When the models were projected to future climates in 2080, lineage I and lineage II were modelled to show a significant northeastward shift of suitable habitats to Northeast China (NEC) and the Russian Far East (Fig. 4c).

## Discussion

The distribution and genetic variation of wild soybean have been significantly shaped by historical climate change. The SSR data resolved wild soybean into two lineages, with lineage I formed by a group of populations from the Yellow River and Huai River valley and lineage II formed by populations from other regions (Figs 1 and 2). The phylogenetic analyses of *trnQ-rps16* failed to detect any deep subdivisions within wild soybean, two commonly haplotypes (H1 and H2) were widely distributed across the range of wild soybean (Fig. 3), and there was no significant geological pattern of genetic and haplotype diversification. The ecological niche modelling analyses suggested the relative narrower distribution of wild soybean during the LGM, which was restricted to central and southern China south of 40°N. There was no suitable habitat modelled in northeastern China, Korea or northern Japan during the LGM, and the present wild soybean populations in these regions probably originated from the northward range shift after the LGM. Both lineages I and II experienced a northward shift after the LGM, though lineage I has expanded on a much smaller scale than lineage II. The large-scale expansion of wild soybean after the LGM is largely consistent with the inferred rapid expansion at approximately 15000 years BP by the BSP analysis (Fig. S1). However, the genetic diversity of wild soybean was not significantly correlated with latitude in northern Eastern Asia (Fig. S3), and multiple endemic plastid haplotypes were detected in NEC, which contrasts with a scenario of a large scale post-glacial northward expansion from southern China, with reduced levels of genetic variation throughout the recolonized regions. We thus could not totally exclude the possibility of the survival of wild soybean in the micro refugia in NEC. Some studies have suggested that mountain glaciers formed only over 2000 metres in the Changbai Mountain region during the late Pleistocene^44^, and lower elevation zones may have had relatively a mild Pleistocene climate and supply microclimatic habitats for biological taxa during glacial periods. Multiple recent phylogeographic studies also suggested refugia in NEC^45^,^46^,^47^.

The geographical pattern of genetic variation of wild soybean was also inferred to be significantly affected by contemporary environmental factors. Traditionally, IBD has been considered a major driver of population divergence^48^. Recently, problems were detected with IBD^49^, and IBE has been considered as a more important driving force for genetic differentiation^50^,^51^,^52^. Recent studies have begun to jointly estimate the relative contribution of these two forces on genetic differentiation at a specific level^15^,^50^,^53^. The comprehensive meta-analysis by Shafer & Wolf^54^ suggested the widespread nature of ecologically induced divergent selection in nature. Some recent studies on different plant species also found that IBE plays a more important role in intraspecific genetic differentiation^15^,^53^. However, IBD was inferred to have a stronger effect than IBE on genetic structure in other plant taxa^24^. The interplay of IBD and IBE in the genetic divergence of species appears to be intricate and system dependent^53^. A stronger effect of IBE versus IBD was found for the genetic differentiation of wild soybean. A Mantel test, partial Mantel test and MMRR analysis all supported the effect of isolation by environmental distance. Multiple ecological processes could shape the pattern of isolation by environment^55^. Wild soybean occurs in diversified habitats across its wide distribution region, and ecological landscape heterogeneity may influence gene flow and connectivity between populations that are adapted to different environments. The PCA analysis showed that temperature and precipitation explain 79.51% of the genetic variation of wild soybean. The Jackknife analysis of ecological niche modelling revealed both precipitation and temperature made a great contribution to model development. All these results indicated that environmental factors played a major role in shaping the genetic structure of the species. Previous studies have suggested the major role of temperature and precipitation in the general adaptation of some other plants^56^.

Integrating the present genetic variation and the contribution of environmental factors to patterns of genetic differentiation, ecological niche modelling of the distribution of biological taxa in past, present and future climates can provide important clues for conserving wild resources. The overlaps between modelled past and present distributions may reveal areas of refugia rich in genetic diversity^57^,^58^. Instead, the lack of overlap between present and predicated future distributions may reveal populations under potential threat from climate change^59^. Both situations will supply clues for conserving wild resources of particular importance and breeding new cultivars adapted to future environmental changes^60^,^61^. Areas of predicted habitat loss should be special targets for *ex situ* conservation in seed banks, botanic gardens, or other germplasm repositories; locations where habitat is likely to be retained may be priorities for *in situ* conservation measures^62^,^63^. Wild soybean was inferred to have a very southern and limited modelled distribution in central and southern China during the LGM, and the modelled suitable habitat will have an obviously northeastward shift in the 2080 s. The present and previous studies have not detected higher population genetic diversification in overlap regions between modelled past and present distributions, and therefore, these areas need not be considered as priority conservation regions. The inferred significant northeastward shift of suitable habitat in the 2080 s suggests that suitable habitat will be lost in the broad region of southern China. At the same time, potential new habitats will be gained, most notably in NEC and the Russian Far East. Large scale *ex situ* conservation measures should be carried out for wild soybean in southern China. The mountain regions of southern China have high micro-geographic environmental heterogeneity, and wild soybean may find suitable habitat through migration over short distances. Therefore, the *ex situ* measures should first consider populations on plains in these regions. Wild soybean usually chooses to live in open habitat, and moderate human disturbance could be beneficial to its establishment and expansion. However, high-density agricultural practices will fragment its habitat. The NEC region is the most concentrated area of agricultural production in China, and many habitats and populations of wild soybean are rapidly diminishing. Comparing large-scale surveys between 1979 to 1983 and 2002 to 2004 revealed large range reductions of wild soybean in this region^64^. Some large populations have disappeared following land conversion for agriculture, which has led to the permanent loss of genotypes, such as the white-flowered soybean type^31^. As the most suitable region for wild soybean in the future, the conservation of wild soybean in this highly disturbed region is not optimistic and the worth of such a project would require further study. Furthermore, environmental factors were inferred to be responsible for the adaptive differentiation of wild soybean, and we should study its local adaptation to new climate conditions for efficient conservation in the face of future climate change.

## Conclusions

Our analyses revealed high genetic variation and differentiation among populations of wild soybean. Wild soybean was inferred to be limited to southern and central China during the LGM, with a large-scale northeastward expansion after the LGM. A significant correlation between genetic distance and environmental distance was identified, which suggested that environmental factors were responsible for the adaptive eco-geographical differentiation of different populations. In combination with genetic studies, the ecological niche modelling of past, present and future distributions is an efficient way to predict geographic regions of high genetic diversity and geographic regions under threat due to future climate change. An urgent area of future study is the possibility for the local adaptation of wild soybean populations to new climate conditions.

## Methods

### Sampling

A total of 604 individuals of wild soybean were collected from 2007 to 2011 in 53 different localities across most of its distribution areas (Table S2, Fig. S4). Individuals separated by at least 50 metres were sampled randomly to avoid collecting ramets from a single genet. Fresh healthy leaves were collected from each sampled individual and dried in silica gel for subsequent DNA extraction. Total DNA was extracted from the dried leaves following the modified CTAB method described by Doyle^65^. The purified total DNA was quantified by gel electrophoresis, and its quality was verified by spectrophotometry. The DNA samples were stored at −20 °C.

### Genotyping of microsatellite loci and cpDNA sequencing

To reduce experimental expenses, genotyping was performed for 43 representive from 53 sampled wild soybean populations using 20 nSSRs, as in previous study (Table S1, He *et al*.^31^). PCR reactions were performed in 15 μL of reaction containing 30–50 ng genomic DNA, 0.6 μM of each primer, 7.5 μL 2 × Taq PCR MasterMix (Transgen, Beijing, China). PCR amplifications were conducted under the following conditions: 94 °C for 2 min; 35 cycles at 94 °C for 30 s, 50 °C for 40 s, and 72 °C for 1 min; followed by a final extension step at 72 °C for 7 min. PCR products were separated on an ABI 3730 DNA sequencer (Applied Biosystems, Foster City, California, USA). Fragment sizes were scored automatically using the program Genemapper (Applied Biosystems).

The plastid *trnQ-rps16* was amplified from 599 individuals representing 52 of 53 populations (we failed to amplify this locus from population J5) using a primer pair of trnQ (GCGTGGCCAAGYGGTAAGGC) and rps16 (GTTGCTTTYTACCACATCGTTT)^66^. *TrnQ-rps16* was amplified and sequenced following the methods of Shaw, *et al*.^66^. The PCR products were purified with an EasyPure PCR Purification Kit (TransGen). Purified PCR products were sequenced directly on an ABI 3730 sequencer.

### Genetic analysis of microsatellite variation

The number of alleles (*A*), the observed heterozygosity (*H*_O_) and expected heterozygosity (*H*_E_) were calculated using GENEALEX v6.4^67^. The polymorphism information content (PIC) was calculated with PowerMaker v3.25^68^ according to Botstein, *et al*.^69^. A hierarchical analysis of molecular variance (AMOVA)^70^ implemented in Arlequin v. 3.11^71^ was used to partition the observed genetic variation among clusters, among populations within a cluster and among individuals within a population.

Genetic differentiation between populations was assessed by the calculation of pairwise *F*_ST_ values among sampling locations, and their significance was calculated with 10,000 permutations implemented in Arlequin v3.11^71^. A dendrogram based on Nei’s standard genetic distance (*D*_nei_)^72^ between populations was constructed using the UPGMA method implemented in PHYLIP v3.68^73^. Genetic differentiation was investigated using the model-based clustering method STRUCTURE v2.1^74^,^75^ for nSSRs. The burn-in time and replication number were set to 100,000 and 100,000 (further generation following the burn in) for each run, respectively. The number of populations (*K*) in the model was systematically varied from 1 to 43. To decrease the margin of error, an average value of 20 simulations performed for each *K* was used. We used the Δ*K* method, representing the highest median likelihood values, to assign wild soybean accessions using the online tool Structure Harvester^76^. For the chosen *K* value, the run that had the highest likelihood estimate was adopted to assign individuals to clusters. The results were visualized using DISTRUCT v1.1^77^.

### Genetic analysis of cpDNA sequence

Gaps (indels) detected in the cpDNA dataset were treated as single mutation events and coded as substitutions (A or T). The haplotype distribution map was constructed using ArcMap v9.3 (ESRI, Redlands, California, USA). A haplotype network was conducted in NETWORK v4.6^78^ using *Glycine tabacina* as an outgroup. A Bayesian Skyline Plot (BSP) in Beast was employed to reconstruct demographic history^79^. This coalescent-based inference method uses a Markov chain Monte Carlo sampling procedure with gene sequence data to estimate a posterior distribution of effective population size through time. To infer the historical demographics of wild soybean, a nucleotide substitution rate of 1.52 × 10^−9^ substitutions per neutral site per year (s/s/y)^80^ was assumed. Markov chains were run for 2.0 × 10^−7^ generations and were sampled every 1,000 generations, with the first 10% being discarded as burn-in.

### Correlations of genetic, geographical and environmental factors

First, the 19 climatic variables of the studied sites were extracted from the WorldClim data set (http://www.worldclim.org/) interpolated to 30-arcsec (ca. 1 km) resolution^81^ using ArcGIS. Then, pairwise Pearson correlations between the 19 factors were calculated. When a pair had a Pearson correlation >0.8, one of the two variables was removed^82^ (Table 4). Finally, seven factors (bio1 = annual mean temperature; bio2 = mean monthly temperature; bio3 = isothemality; bio4 = temperature seasonality; bio5 = max temperature of warmest month; bio13 = precipitation of wettest month; bio15 = precipitation seasonality) were chosen as representative of climate factors.

The Mantel test^83^ was used to detect the correlation between pairwise Nei’s distance vs. pairwise geographical distance and pairwise Nei’s distance vs. pairwise environmental distance. Matrices of pairwise Nei’s distance and pairwise geographical distance were generated with GenAlEx v6.5^84^. The environmental distance was calculated in NTSYSPC v2.11c^85^ using the seven identified factors. The Mantel test was performed with program zt^86^ and 10,000 permutations were used in significance testing.

The correlation between genetic differentiation and geographical/environmental factors were determined by a combination of a partial Mantel test^87^ and a matrix regression analysis^88^ using the above distance matrices. A partial Mantel test was performed with program zt^86^, and 10,000 permutations were used in significance testing. Multiple matrix regression with randomization (MMRR) is a novel and robust approach for estimating the independent effects of potential factors^89^,^90^, and the analysis was implemented with 10,000 permutations in R with the MMRR function script^88^.

### Ecological niche modelling (ENM)

Ecological niche modelling was carried out in MAXENT v3.3.3^43^,^91^ to predict the geographic distribution of climatically suitable habitats for wild soybean. MAXENT calculates probability distributions based on incomplete information and does not require absence data, making it appropriate for modelling species distributions based on presence-only herbarium records^43^. The sampling sites of 43 populations in combination with 175 presence records obtained from the Chinese Virtual Herbarium (http://www.cvh.org.cn/cms/cn) were included in this study (Table S2, Fig. S4). We employed the 8 aforementioned bioclimatic variables to implement this model. Most of the default parameters of MAXENT were used to conduct ENM, except the following user-selected parameters: application of random seed and random test percentage of 70%, replicates of 10 and bootstrap as the replicated run type. The logistic output of MAXENT consists of a grid map with each cell having an index of suitability between 0 and 1. Low values indicate that conditions are unsuitable for the species, whereas high values indicate that conditions are suitable. Model predictions were visualized in ARCMAP v9.3 (ESRI, Redlands, CA).

To obtain the distribution of wild soybean at the Last Glacial Maximum, we projected present species-climate relationships to the LGM using the Model for Interdisciplinary Research on Climate (MIRIC v3.2)^92^ scaled down to a 2.5-arcmin resolution. To explore the importance of each predictor, we carried out Jackknife analyses of the regularized gain using training data. To clarify the possible demographic history of two different lineages (see results), we analysed each of their distributions in the LGM.

To model the suitability of wild soybean in future climates, we applied one commonly used general circulation model, the Model for Interdisciplinary Research on Climate (MIRIC). The ecological niche modelling predicted with present climatic variables was projected on the global circulation model for the year 2080. The performance of the model prediction was evaluated using the area under the (receiver operation characteristic) curve (AUC) calculated by MAXENT.

## Additional Information

**How to cite this article**: He, S. *et al*. Environmental and Historical Determinants of Patterns of Genetic Differentiation in Wild Soybean (*Glycine soja* Sieb. et Zucc). *Sci. Rep.* **6**, 22795; doi: 10.1038/srep22795 (2016).

## Supplementary Material

Supplementary Information

## Figures and Tables

**Table 1:** Genetic diversity parameters estimated by 20 nSSRs in 43 populations of wild soybean.

Pop.	A	*H*_O_	*H*_E_	PIC	Pop.	A	*H*_O_	*H*_E_	PIC
AF	3.2	0.014	0.397	0.359	JZ	2.2	0.018	0.455	0.357
HY	4.4	0.014	0.605	0.562	QH	2.5	0.010	0.212	0.195
JO	2.3	0.007	0.300	0.260	WQ	4.1	0.003	0.555	0.513
QZ	2.8	0.015	0.456	0.387	XH	2.4	0.000	0.367	0.310
RY	1.8	0.018	0.094	0.088	YT	1.2	0.005	0.037	0.030
DQ	2.9	0.051	0.468	0.407	HL	5.1	0.037	0.658	0.614
SC	5.7	0.023	0.722	0.687	JH	5.4	0.082	0.694	0.649
TB	6.8	0.007	0.797	0.769	KS	3.1	0.003	0.582	0.513
WC	6.2	0.053	0.762	0.729	LX	3.4	0.028	0.520	0.468
XU	5.7	0.205	0.708	0.665	QQ	3.9	0.003	0.504	0.460
CK	2.9	0.037	0.491	0.427	SY	1.3	0.061	0.076	0.061
CY	1.9	0.013	0.200	0.173	J1	3.2	0.021	0.563	0.506
GH	3.1	0.048	0.426	0.379	J2	1.2	0.007	0.018	0.017
N1	3.0	0.027	0.383	0.342	J3	1.2	0.003	0.064	0.053
N2	2.6	0.067	0.407	0.332	J4	3.2	0.034	0.467	0.048
YJ	2.6	0.014	0.414	0.341	J5	2.9	0.069	0.45	0.393
BX	4.9	0.028	0.570	0.533	K1	1.5	0	0.09	0.082
HX	4.9	0.071	0.598	0.560	K2	1.3	0.013	0.023	0.022
LW	2.0	0.007	0.265	0.225	K3	1.7	0	0.153	0.133
WS	3.8	0.017	0.520	0.473	K4	6.3	0.092	0.708	0.673
YL	2.4	0.010	0.302	0.260	K5	4.5	0.1	0.711	0.660
DY	3.4	0.018	0.526	0.470	mean	3.3	0.031	0.426	0.373

*A*: number of alleles; *A*_R_: allele richness; *H*_O_: observed heterozygosity; *H*_E_: expected heterozygosity; PIC: polymorphism information content.

**Table 2:** Analysis of molecular variance (AMOVA) for wild soybean.

Loci	Source of variation	SS	VC	PV(%)	Fixation indices
nSSR	Among two lineage	393.04	0.565	5.99	*F*_CT_ = 0.006
	Among populations within lineage	5106.23	4.409	46.69	*F*_ST_ = 0.527
	Within populations	5050.64	4.469	47.32	*F*_SC_ = 0.497

**Table 3:** Results of the Mantel test, partial Mantel test and MMRR analysing the correlation between geographical distances, environmental distances and Nei’s genetic distance based on microsatellite data.

	Mantel test		partial Mantel test		MMRR	
	*r*	*P* value	*r*	*P* value	*β*	*P* value
Gen. Geo	−0.016	0.341	−0.002	0.508	0.006	0.294
Gen. Env	0.233	**0.002**	0.232	**0.001**	0.205	**0.025**

Regular letters refer to non-significant results and bold letters refer to significant correlations.

Geo, geographical distance; Gen, genetic distance; Env, environmental distance.

**Table 4:** Multi-collinearity test using cross-correlations (Pearson correlation coefficients, *r*) among environmental variables.

Variables	Bio1	Bio2	Bio3	Bio4	Bio5	Bio6	Bio7	Bio8	Bio9	Bio10	Bio11	Bio12	Bio13	Bio14	Bio15	Bio16	Bio17	Bio18	Bio19
Bio1																			
Bio2	−0.717																		
Bio3	−0.023	0.428																	
Bio4	−0.613	0.373	−0.652																
Bio5	0.673	−0.502	−0.615	0.155															
Bio6	0.944	−0.735	0.169	−0.817	0.417														
Bio7	−0.712	0.565	−0.485	0.974	0.026	−0.898													
Bio8	0.570	−0.455	−0.567	0.193	0.865	0.334	0.052												
Bio9	0.932	−0.659	0.249	−0.834	0.384	0.983	−0.895	0.298											
Bio10	0.761	−0.612	−0.576	0.044	0.982	0.528	−0.105	0.887	0.493										
Bio11	0.930	−0.643	0.282	−0.860	0.363	0.991	−0.914	0.282	0.989	0.471									
Bio12	0.661	−0.701	−0.016	−0.578	0.293	0.734	−0.665	0.173	0.699	0.377	0.705								
Bio13	0.423	−0.458	0.062	−0.435	0.082	0.487	−0.496	0.085	0.477	0.179	0.480	0.831							
Bio14	0.687	−0.714	−0.202	−0.416	0.496	0.695	−0.524	0.317	0.655	0.547	0.651	0.912	0.638						
Bio15	−0.663	0.736	0.129	0.509	−0.394	−0.722	0.603	−0.227	−0.674	−0.444	−0.676	−0.825	−0.425	−0.862					
Bio16	0.498	−0.531	0.114	−0.552	0.078	0.589	−0.610	0.018	0.574	0.179	0.582	0.905	0.969	0.709	−0.555				
Bio17	0.701	−0.725	−0.200	−0.423	0.503	0.707	−0.534	0.316	0.668	0.557	0.662	0.913	0.637	0.995	−0.873	0.712			
Bio18	0.369	−0.408	0.176	−0.501	−0.036	0.478	−0.543	0.060	0.462	0.067	0.480	0.816	0.948	0.598	−0.456	0.927	0.593		
Bio19	0.714	−0.715	−0.179	−0.436	0.503	0.715	−0.542	0.293	0.693	0.557	0.675	0.920	0.666	0.980	−0.852	0.745	0.982	0.593	
