# Supplementary material for: Environmental and Historical Determinants of Patterns of Genetic Differentiation in Wild Soybean (Glycine soja Sieb. et Zucc)
Source: Sci Rep. 2016 Mar 8;6:22795. doi: 10.1038/srep22795 (PMC4782138; doi:10.1038/srep22795)
Supplement: Supplementary Information [file srep22795-s1.pdf]

Environmental and Historical Determinants of Patterns of Genetic Differentiation in Wild Soybean (*Glycine soja* Sieb. et Zucc)

Shuilian He, Yunsheng Wang, Dezhu Li & Tingshuang Yi

Supporting Information

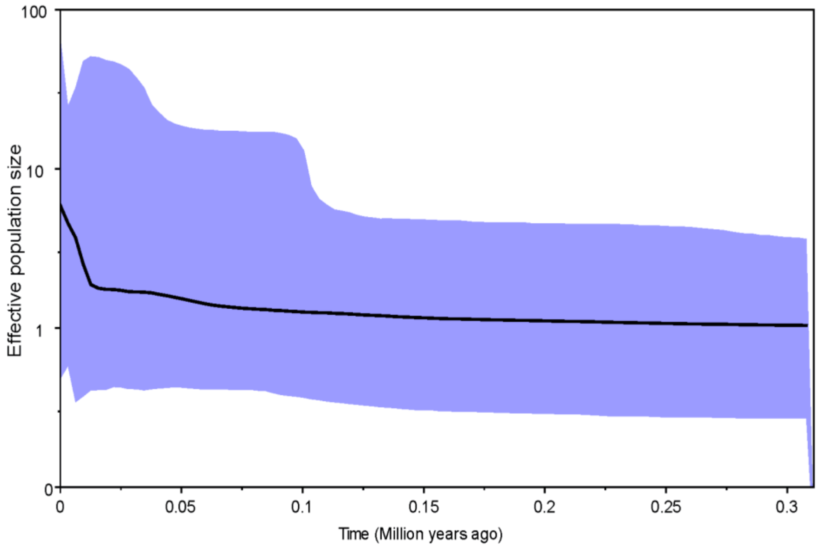

Figure S1 The effective population size over time for (a) all 52 populations of wild soybean.

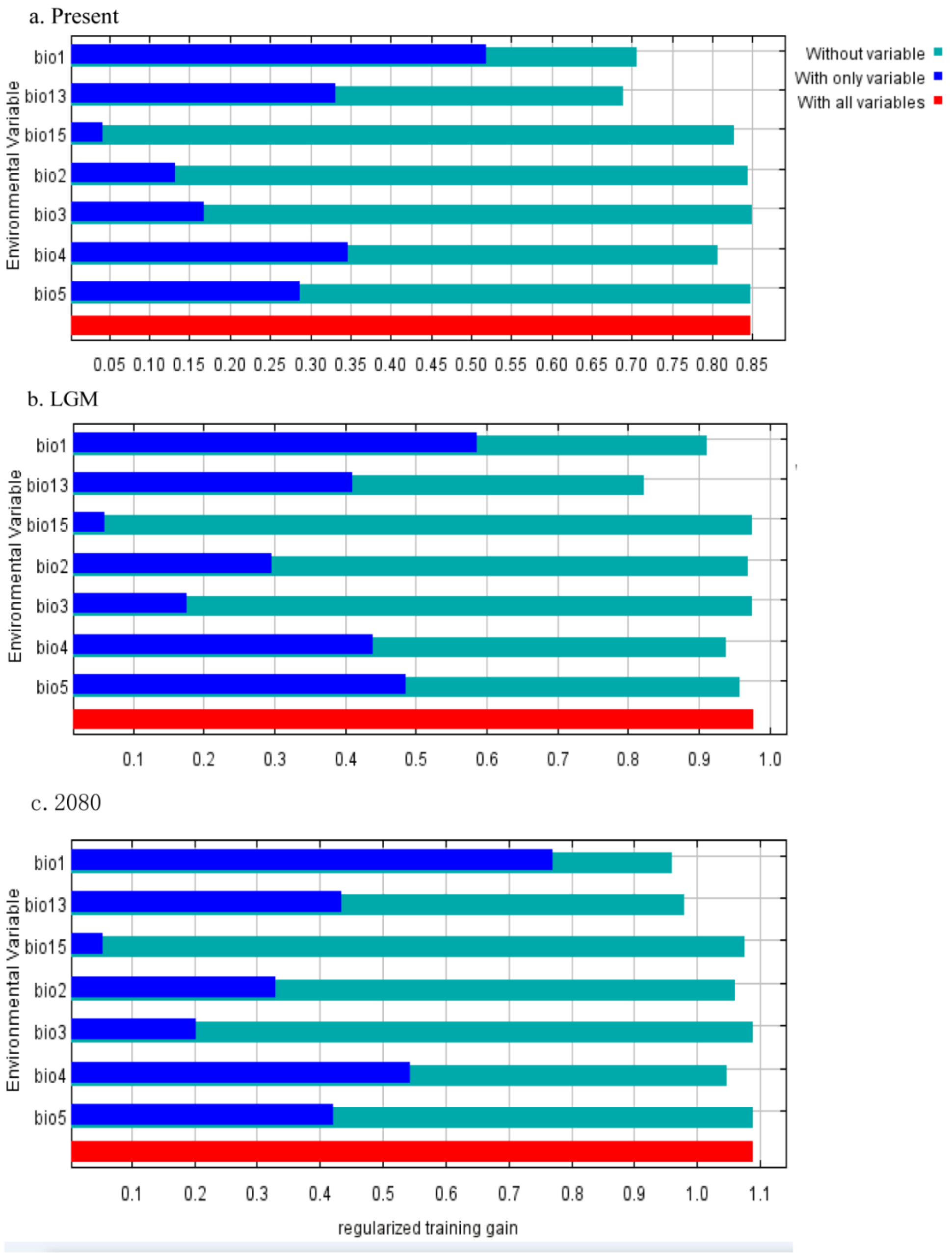

Figure S2 Jackknife analyses of individual predictor importance for wild soybean applied to the Maxent model in relation to overall model quality or "total gain" (grid bar). Dark blue bars indicate the gain achieved when including that predictor only and excluding remaining predictors; light blue show how the total gain is diminished without the given predictor.

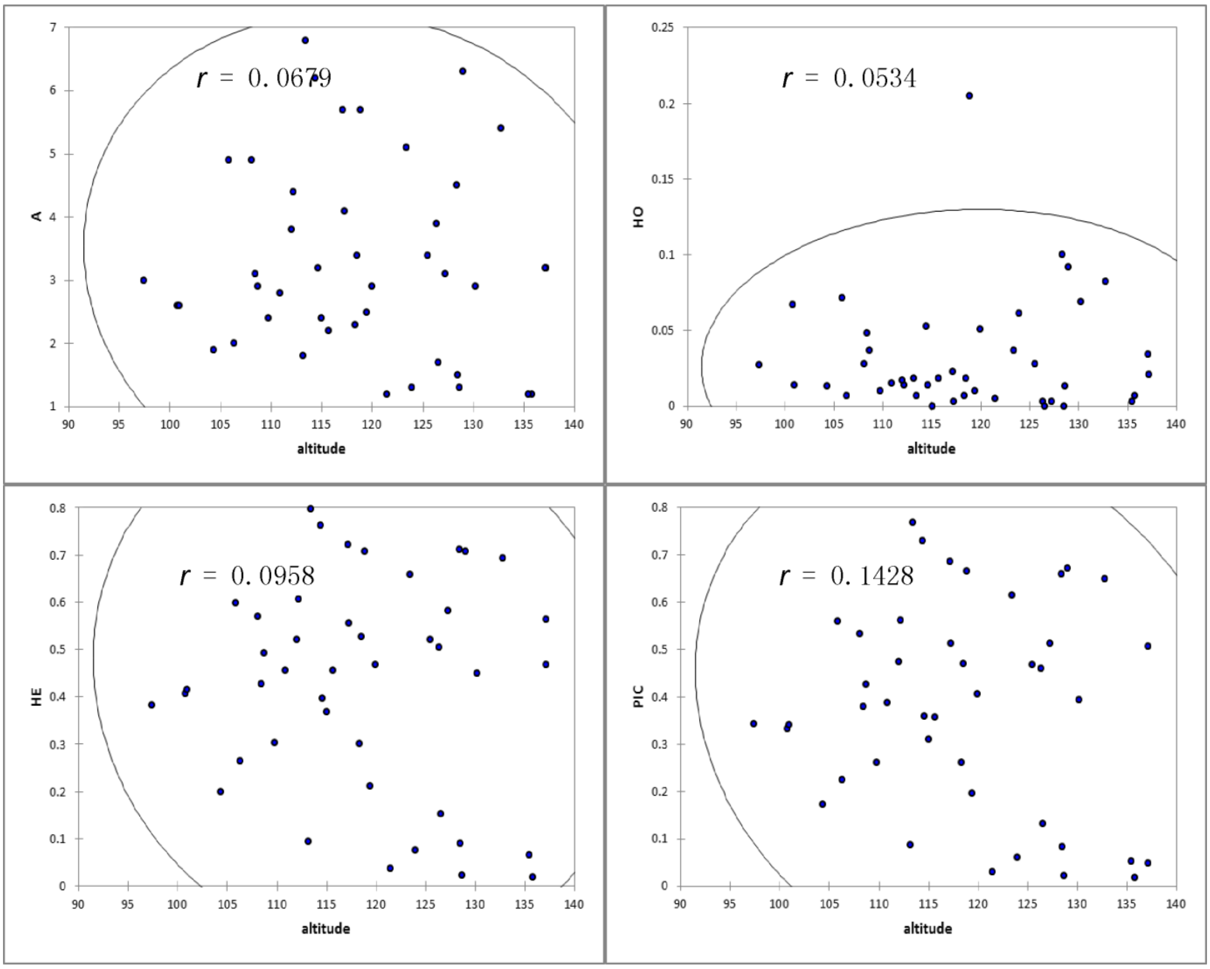

Figure S3Correlation analysis between altitude and genetic diversity.

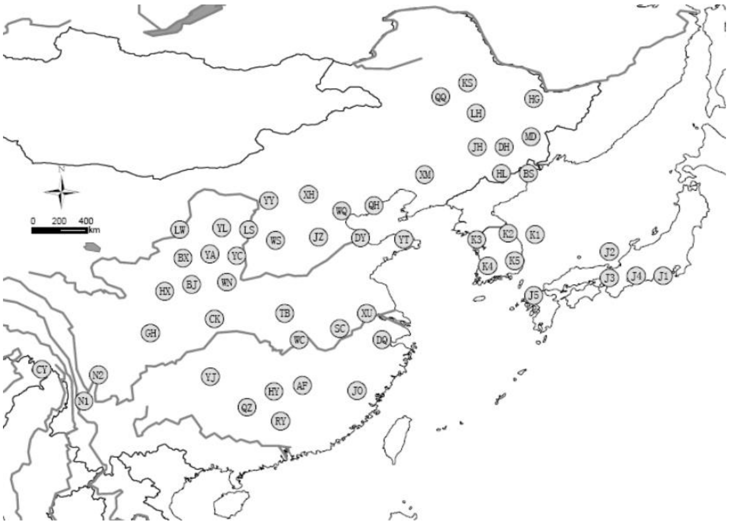

Figure S4 Sample locations of 53 wild soybean populations (ArcMap v9.3)**.**

Table S1 Primers and genetic characteristic of the SSR markers used to analyze the collection. For each of the 20 markers, the primer sequence, repeat motif, size range, chromosomal location, number of alleles and genotypes, polymorphism information content (PIC) value and neutral test results are presented. neu: neutural loci; non: non-neutral test.

Primer name	Primer sequence(5'to3')	Repeat motif	Size range	linkage group	Allele no.	Genotype no.	PIC	Neutral test
Satt126	F:ATAAAACAAATTCGCTGATAT<br>R:GCTTGGTAGCTGTAGGAA	(ATT)18	109-172	B2	16	22	0.788	neu
Satt135	F:TTCCAATACCTCCCAACTAAC<br>R:CACGGATTTTAAATCATTATTACAT	(ATT)19	141-204	D2	13	19	0.773	neu
satt215	F:GCGCCTTCTTCTGCTAAATCA<br>R:CCCATTCAATTGAGATCCAAAATTAC	(ATT)11	114-221	J	23	34	0.812	neu
Satt216	F:TACCCTTAATCACCGGACAA<br>R:AGGGAACTAACACATTTAATCATCA	(ATT)20	137-251	D1b	24	41	0.909	non
Satt221	F:GCGGCAAACCATTATCTTCATT<br>R:GCGATTGTACCACTAAAAACCATAG	(ATT)23	109-224	D1a	36	44	0.940	non
Satt231	F:GGCACGAATCAACATCAAAACTTC<br>R:GCGTGTGCAAAATGTTCATCATCT	(ATT)32	160-328	E	36	47	0.934	non
satt233	F:AAGCATACTCGTCGTAAC<br>R:GCGGTGCAAAGATATTAGAAA	(ATT)16	169-238	A2	21	33	0.863	neu
Satt270	F:TGTGATGCCCCTTTTCT<br>R:GCGCAGTGCATGGTTTTCTCA	(ATT)16	183-249	I	18	22	0.764	neu
satt277	F:GGTGGTGGCGGGTTACTATTACT<br>R:CCACGCTTCAGTTGATTCTTACA	(ATT)40	128-255	C2	32	45	0.935	non
satt288	F:GCGGGGTGATTTAGTGTTTGACACCT<br>R:GCGCTTATAATTAAGAGCAAAAGAAG	(ATT)17	195-273	G	25	46	0.901	non

Table S1 continue

Primer name	Primer sequence(5'to3')	Repeat motif	Size range	linkage group	Allele no.	Genotype no.	PIC	Neutral test
Satt294	F:GCGCTCAGTGTGAAAGTTGTTTCTAT<br>R:GCGGGTCAAATGCAAATTATTTTT	(ATT)23	237-303	C1	18	26	0.902	non
Satt373	F:TCCGCGAGATAAATTCGTAAAAT<br>R:GGCCAGATACCCAAGTTGTACTTGT	(TAT)21	210-279	L	23	35	0.914	non
Satt423	F:TTCGCTTGGGTTCAGTTACTT<br>R:GTTGGGGAATTAAAAAAATG	(ATT)19	225-351	F	24	37	0.878	neu
Satt463	F:CTGCAAATTTGATGCACATGTGTCTA<br>R:TTGGATCTCATATTCAAACTTTCAAG	(ATT)19	100-214	M	29	31	0.917	non
satt509	F:GCGCAAGTGGCCAGCTCATCTATT<br>R:GCGCTACCGTGTGGTGGTGTGCTACCT	(ATT)30	119-242	B1	22	32	0.921	non
Satt530	F:CCAAGCGGGTGAAGAGGTTTTT<br>R:CATGCATATTGACTTCATTATT	(ATT)12	201-279	N	25	34	0.916	non
satt555	F:GCGGTTGGCTTTGATGATGT<br>R:TTACCGCATGTTCTTGGACTA	(ATT)13	234-312	K	20	33	0.889	non
Satt568	F:CGGACACCGGTCTACTAGGAAAGTAA<br>R:GCGGAATAATCCAATTCAATTTA	(ATT)17	212-275	H	19	28	0.903	non
satt572	F:GCGGAGCATGTAAATCCAGCCTATTGA<br>R:GCGGGCTAACTTATGTTACTAAACAAT	(ATT)14	130-241	A1	27	36	0.934	non
satt581	F:CCAAAGCTGAGCAGCTGATAACT<br>R:CCCTCACTCCTAGATTATTTGTTGT	(ATT)11	130-196	O	16	32	0.870	non
mean					23	34	0.883	

Table S2 The sampling locality, longitude, latitude, altitude and habitate of 53 wild soybean populations

Pop.	Location	Latitude (N)	Longitude (E)	Altitude (m)	sampling
AF	Anfu county, Jiangxi province	27°23′17″	114°36′ 06″	85	Seq, SSR
JO	Jianou county, Fujian province	27°01′ 28″	118°17′ 34″	126	Seq, SSR
HY	Hengyang county, Hunan province	26°57′ 43″	112°09′ 12″	123	Seq, SSR
RY	Ruyuan county, Guangdong province	24°55′ 12″	113°08′ 12″	510	Seq, SSR
QZ	Quanzhou county, Guangxi Zhuang province	25°52′ 21″	110°51′ 43″	722	Seq, SSR
WC	Wuchang district, Hubei province	30°31′ 16″	114°23′ 44″	15	Seq, SSR
XU	Xuanwu district, Jiangsu province	32°02′ 44″	118°51′ 39″		Seq, SSR
WQ	Duqing county, Zhejiang province	30°33′ 00″	119°58′ 20″	15	Seq, SSR
SC	Shucha county, Anhui province	31°18′ 51″	117°07′ 40″	45	Seq, SSR
TB	Tongbai county, Henan province	32°22′ 12″	113°24′ 01″	33	Seq, SSR
CK	Chengkou county, Chongqing	31°58′ 98″	108°40′ 02″	805	Seq, SSR
YJ	Yinjiang county, Guizhou province	28°.00′ 03″	108°24′ 36″	458	Seq, SSR
GH	Guanghan city, Sichuan province	30°59′ 49″	104°20′ 55″	458	Seq, SSR
CY	Chayu county, Xizang province	27°03′ 53″	100°50′ 03″	1685	Seq, SSR
N1	Ninglang county, Yunnan province	28°30′ 08″	97°00′ 38″	2600	Seq, SSR
N2	Ninglang county, Yunnan province	28°06′ 54″	99°51′ 02″	2550	Seq, SSR
BX	Bingxian county, Shaanxi province	35°02′ 26″	108°04′ 39″	835	Seq, SSR
HX	Huixian county, Gansu province	33°53′ 36″	105°49′ 33″	1126	Seq, SSR
YY	Youyu county, Shanxi province	40°08′ 38″	112°20′ 22″	1279	Seq
LW	Lingwu county, Ningxia province	38°08′ 44″	106°19′ 33″	1103	Seq, SSR
WS	Wenshui county, Shaanxi province	37°25′ 02″	112°01′ 02″	759	Seq, SSR
YA	Yan’an county, Shaanxi province	36°37′ 29″	109°27′ 18″	962	Seq
YL	Yulin city, Shaanxi province	38°16′ 50″	109°44′ 18″	1051	Seq, SSR
LS	Linxian county, Shaanxi province	37°56′ 51″	110°59′ 15″	948	Seq
YC	Yichuan county, Shaanxi province	36°03′ 31″	110°11′ 20″	829	Seq
BJ	Baoji city, Shaanxi province	34°22′ 04″	107°0′ 05″	586	Seq
JZ	Jizhou county, Hebei province	37°44′ 31″	115°41′ 12″	23	Seq, SSR
WN	Weinan city, Shaanxi province	34°31′ 01″	109°30′ 00″	355	Seq
DY	Dongying city, Shandong province	37°34′ 25″	118°31′ 27″	6	Seq, SSR
DQ	Wuqing district, Tianjing	39°26′ 57″	117°14′ 57″	-6	Seq, SSR

Table S2 continue

Pop.	Location	Latitude (N)	Longitude (E)	Altitude (m)	sampling
XH	Xuanhua county, Hebei province	40 °35′ 34″	115 °01′ 17″	601	Seq, SSR
QH	Qinghuangdao city, Hebei province	39 °48′ 29″	119 °25′ 54″	18	Seq, SSR
YT	Yantai city, Shandong province	37 °28′ 65″	121 °27′ 14″	10	Seq, SSR
LX	Lanxi county, Heilongjiang province	46 °13′ 06″	126 °20′ 16″	139	Seq, SSR
JH	Jiaohe county, Jinlin province	43 °48′ 28″	127 °14′ 15″	126	Seq, SSR
SY	Xinming county, Liaoning province	41 °57′ 19″	122 °51′ 29″	28	Seq, SSR
MD	Mudanjiang city, Heilongjiang province	44 °34′ 07″	129 °36′ 26″	253	Seq
BS	Baishan city, Jilin province	42 °27′ 00″	128 °07′ 44″	701	Seq
KS	Keshan county, Heilongjaing province	48 °17′ 00″	125 °29′ 54″	325	Seq, SSR
QQ	Qiqihaer city, Heilongjiang province	47 °20′ 27″	123 °56′ 24″	304	Seq, SSR
HG	Hegan city, Heilongjiang province	47 °12′ 20″	130 °13′ 46″	213	Seq
DH	Dunhua city, Jilin province	43 °22′ 28″	128 °13′ 36″	495	Seq
HL	Hulin city, Heilongjiang province	42 °02′ 24″	128 °03′ 16″	73	Seq, SSR
J1	Kanagawa, Japan	34 °57′ 33″	137 °08′ 20″	12	Seq, SSR
J2	Tokyo, Japan	34 °49′ 41″	135 °46′ 13″	35	Seq, SSR
J3	Hirakata,Osaka,Japan	34 °48′ 52″	135 °29′ 02″	11	Seq, SSR
J4	Okazaki, Japan	34 °57′ 33″	137 °08′ 20″	37	Seq, SSR
J5	Kyushu University, Fukuoka, Japan	33 °35′ 47″	130 °12′ 55″	48	Seq, SSR
K1	Gangwon-do, South Korea	37 °35′ 15″	128 °24′ 33″	520	SSR
K2	Gangwon-do, South, Korea	37 °54′ 47″	128 °29′ 56″	340	Seq, SSR
K3	Incheon, South Korea	37 °27′ 30″	126 °21′ 53″	11	Seq, SSR
K4	Yeongcheon-si city, Korea	37 °54′ 47″	128 °29′ 56″	102	Seq, SSR
K5	Moonkyeong-si city, Korea	37 °27′ 30″	126 °21′ 53″	77	Seq, SSR
